# Supplementary material for: A deep learning framework deploying segment anything to detect pan-cancer mitotic figures from haematoxylin and eosin-stained slides
Source: Commun Biol. 2024 Dec 19;7:1674. doi: 10.1038/s42003-024-07398-6 (PMC11659629; doi:10.1038/s42003-024-07398-6)
Supplement: Supplementary file 2 — Supplementary Information [file 42003_2024_7398_MOESM2_ESM.pdf]

A Deep Learning Framework Deploying Segment Anything to Detect Pan-Cancer Mitotic Figures from Haematoxylin and Eosin-Stained Slides

Zhuoyan Shen[1*], Mikael Simard[1], Douglas Brand[1, 2], Vanghelita Andrei[3, 4], Ali Al-Khader[3, 4], Fatine Oumlil[4], Katherine Trevers[3, 4], Thomas Butters[3], Simon Haefliger[3,5], Eleanna Kara[6], Fernanda Amary[3, 4], Roberto Tirabosco[3, 4], Paul Cool[7,8], Gary Royle[1], Maria A. Hawkins[1,2], Adrienne M. Flanagan[3,4†], Charles-Antoine Collins Fekete[1†]

1. Department of Medical Physics and Biomedical Engineering, University College London, London, United Kingdom.
2. Department of Radiotherapy, University College London Hospitals NHS Foundation Trust, London, United Kingdom.
3. Research Department of Pathology, University College London Cancer Institute, London, United Kingdom.
4. Cellular and Molecular Pathology, Royal National Orthopaedic Hospital NHS Foundation Trust, Middlesex, United Kingdom.
5. Institute of Medical Genetics and Pathology, University Hospital Basel, University of Basel, Basel, CH, Switzerland.
6. Department of Neurology, Rutgers Biomedical and Health Sciences, Rutgers University, New Jersey, United States.
7. Department of Orthopaedics, The Robert Jones and Agnes Hunt Orthopaedic Hospital, Oswestry, United Kingdom.
8. School of Medicine, Keele University, Newcastle, United Kingdom.

†: These authors jointly supervised this work

*: Corresponding author

Supplementary Information

Figures

Supplementary Figure 1: Figures with disagreement between pathologists. The figures indicated by the yellow arrows were the examples labelled as “equivocal” by the junior pathologists and were sent to senior pathologists for secondary annotating.

Supplementary Figure 2: Low-quality masks. Examples of mitotic figure (MF) masks (the blue masks) that are badly delineated by Segment Anything (SAM). The MFs are indicated by yellow arrows, the green boxes are the prompts used when deploying the SAM.

Supplementary Figure 3: Example images showing the nuclei segmented by the original and fine-tuned Segment Anything (SAM). Images are from the Lizard dataset with the ground truth nuclei contours (in green) manual annotated by pathologists.

Tables

Supplementary Table 1. Classification metrics of different classifiers tested for mitotic figure classification.

Supplementary Table 2. The DICE coefficient of different models on segmenting nuclei in the Lizard dataset.

Supplementary Table 3. Summary of datasets and scanners.

Supplementary Table 4. Number of whole slide images (WSIs) and mitotic figures (MFs) for each diagnosis in STMF.

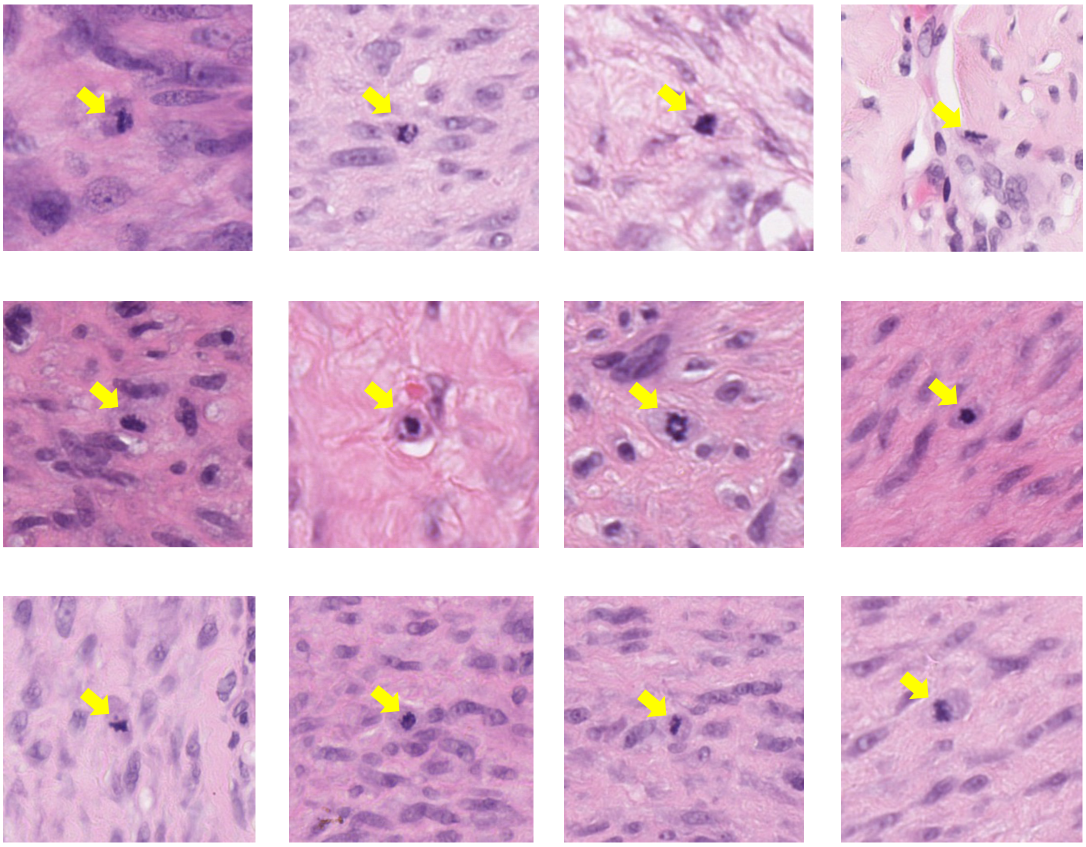

Supplementary Figure 1: Figures with disagreement between pathologists. The figures indicated by the yellow arrows were the examples labelled as “equivocal” by the junior pathologists and were sent to senior pathologists for secondary annotating.

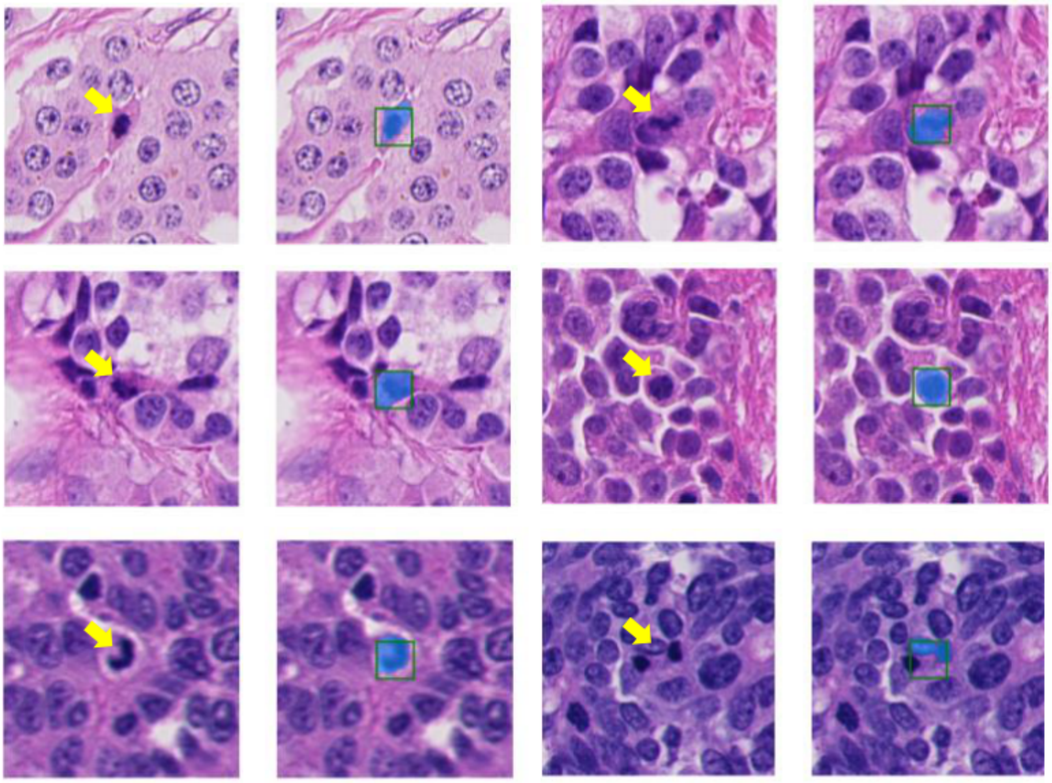

Supplementary Figure 2: Low-quality masks. Examples of mitotic figure (MF) masks (the blue masks) that are badly delineated by Segment Anything (SAM). The MFs are indicated by yellow arrows, the green boxes are the prompts used when deploying the SAM.

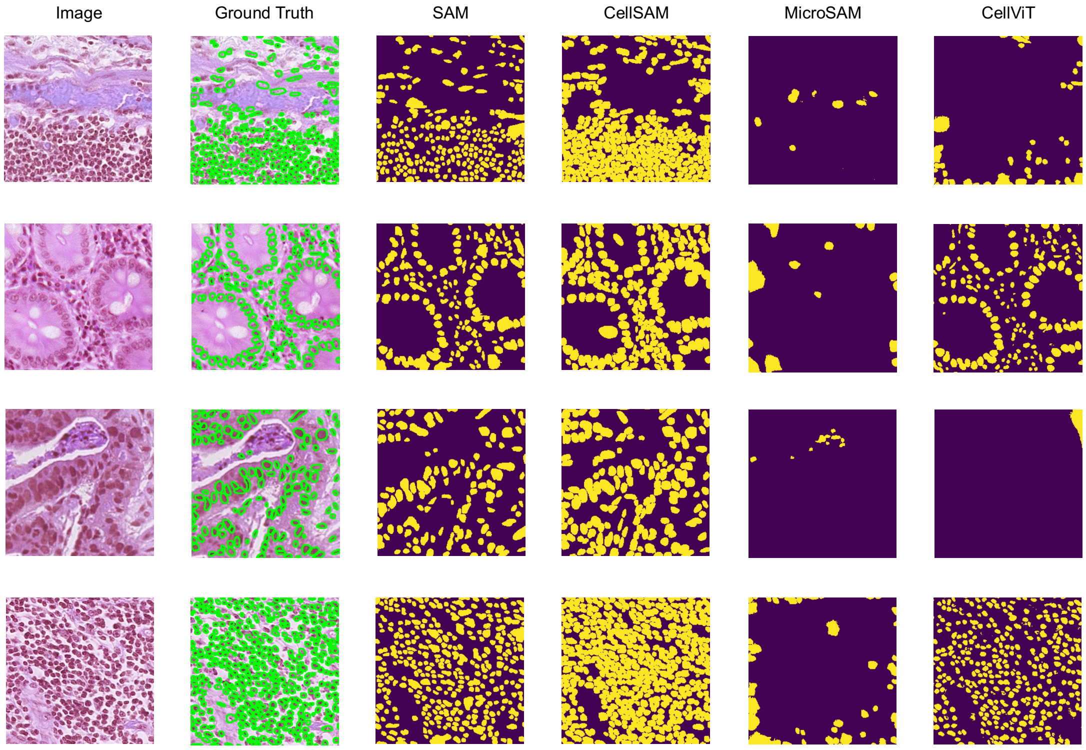

Supplementary Figure 3: Example images showing the nuclei segmented by the original and fine-tuned Segment Anything (SAM). Images are from the Lizard dataset with the ground truth nuclei contours (in green) manual annotated by pathologists.

Supplementary Table 1. Classification metrics of different classifiers tested for mitotic figure classification.

Classifier	Precision	Recall	F1	Accuracy	AUC
ResNet18	**0.851**	0.842	**0.846**	**0.995**	**0.949**
ResNet50	0.842	0.836	0.838	0.995	0.928
DenseNet121	0.834	0.820	0.827	0.994	0.924
ConvNeXt-tiny	0.839	**0.845**	0.842	0.994	0.946
Efficient-Net-B7	0.841	0.840	0.841	0.994	0.941

Supplementary Table 2. The DICE coefficient of different models on segmenting nuclei in the Lizard dataset.

	SAM	CellViT	CellSAM	MicroSAM
DICE	0.76 ± 0.13	0.48 ± 0.34	0.38 ± 0.15	0.25 ± 0.13

Supplementary Table 3. Summary of datasets and scanners

Dataset	Scanners
ICPR	Aperio Scanscope XT<br>Hamamatsu Nanozoomer 2.0-HT
TUPAC	Leica SCN400
MIDOG++	Hamamatsu XR<br>Hamamatsu S360<br>3DHistech Pannoramic Scan II<br>Aperio ScanScope CS2
CMC	Aperio ScanScope CS2
CCMCT	Aperio ScanScope CS2
STMF	Aperio ScanScope CS2

Supplementary Table 4. Number of whole slide images (WSIs) and mitotic figures (MFs) for each diagnosis in STMF.

Diagnosis	Number of WSIs	Number of MFs
Angiosarcoma	4	672
Chondrosarcoma	3	105
Ewing Sarcoma	3	54
Giant Cell Tumour of bone	3	195
Leiomyosarcoma	12	381
Solitary Fibrous Tumour	99*	652
Dedifferentiated liposarcoma	1	54
Melanoma	3	411
Myeloma	1	44
Myxofibrosarcoma	10	826
Nerve Sheath Tumour	1	49
MPNST	1	88
Osteosarcoma	9	130
Pleomorphic Sarcoma	30	2228
Rhabdomyosarcoma	3	66
NFATC2-Sarcoma	1	43
Spindle Cell Sarcoma	10	1326
Synovial Sarcoma	7	502
Desmoid fibromatosis	74*	34
Superficial fibromatosis	53*	37

*Annotated by active learning. The other data is produced by pHH3 staining.
